# No Backstage: The Relentless Emotional Management of Acute Nursing Through the COVID‐19 Pandemic

**DOI:** 10.1111/jan.16563

**Published:** 2024-10-24

**Authors:** Aileen Grant, Rosaleen O'Brien, Flora Douglas, Catriona Kennedy, Debbie Baldie, Nicola Torrance

**Affiliations:** ^1^ School of Nursing Midwifery and Paramedic Practice Robert Gordon University Aberdeen Scotland; ^2^ Independent Scholar Glasgow UK; ^3^ NHS Grampian Scotland

**Keywords:** burnout, compassion, COVID‐19 pandemic, emotional intelligence, nurse–patient interaction, organisational behaviour, pandemic, patient safety, teamwork, work organisation

## Abstract

**Aim(s):**

To explore the impact of the COVID‐19 pandemic on nurse's well‐being, experiences of delivering healthcare within acute settings and their emotional management.

**Design:**

Sequential mixed methods.

**Methods:**

February to July 2021 an online well‐being survey was disseminated to nurses working in acute settings within one Scottish health board. In‐depth interviews with a purposive sample of respondents were conducted. Survey data were analysed descriptively, and interview data using Framework analysis and emotional management as the theoretical framework.

**Results:**

Well‐being was poor overall. Infection control measures impeded interactions, with loss of connection between patients, families and nurses. Emotional work was extended in caring for patients and families when visits were forbidden or restricted. Disconnect between colleagues was intensely felt. On COVID and non‐COVID wards, nurses were caring for patients with a significantly reduced workforce and often outside their clinical speciality. Nurses masked their own anxieties, fears, moral distress and exhaustion on the ward. Communal ‘backstage’ spaces, were reduced to enable more infection‐control space but reduced opportunity for collegial support. Formal psychological intervention required access after shift, and/or nurses feared they could not contain their emotions afterwards.

**Conclusion:**

Working during the pandemic was emotionally and physically demanding for those in COVID a.nd non‐COVID wards. Unintended consequences of infection control measures significantly extended nurses' emotional management, by caring for isolated patients and families but impeding opportunities to care for each other, compounding their emotions.

**Implications for the Profession:**

There is a need to value emotional work in nursing to better support mental well‐being.

**Impact:**

We advance the nursing emotional management literature by addressing the gap of exploration in challenging conditions. The importance of emotional management on nurses' mental well‐being has been overlooked but focusing on this in the next crisis could improve nurse's well‐being.

**Patient or Public Contribution:**

No patient or public contribution.

**Reporting Method:**

GRAMMS.

**Summary**

- What is already known?
  ○ The COVID‐19 pandemic brought challenges for healthcare staff, including difficult working conditions, depleted care standards and conditions, moral distress, emotional exhaustion and burnout.
  ○ Exacerbating problems within the nursing workforce pre‐pandemic.
- What this paper adds?
  ○ Nursing work through the early waves of the COVID pandemic was relentless and emotionally demanding. Nurses experienced a wide range of emotions, including fear, moral distress and isolation. Working conditions were challenging due to rapid reorganisation of ways of working, wearing PPE, social distancing measures and the number of people contracting COVID‐19 rising. It was a complex and challenging situation, where nurses frequently experienced conflicting values and beliefs.
  ○ The setting (stage) was different, working in isolation or with people they had not worked with before and observing increased suffering and death with a new disease with which very little was known and with no end in sight. It was difficult to alleviate distress in patients and families when wearing masks. This required more effort in their emotional management performance when interacting with patients, families and colleagues.
  ○ Emotional well‐being was poor with 45% of respondents reporting symptoms of depression, further adding to the effort required to perform. Managing their emotions was difficult without their usual strategies. Almost all staff missed usual backstage support mechanisms, such as humour, hugs and conversations about difficult situations. Formal psychological interventions were put in place, but only accessed by a minority of respondents. There was a culture of being stoic and some feared stigma engaging in psychological services.
- Implications for policy and practice
  ○ There is a need for recognition of the work of emotional management.
  ○ During the pandemic, focus was on infection control measures rather than how these measures affected interactions between nurses, patients and families. These caring interactions should be prioritised in future. In particular, recognition of the shared understanding and emotional support between colleagues.
  ○ There is a need to dedicate time to off stage interaction in caring for one another, especially in times when frontstage performances are harder to perform.

## 1 Introduction

The COVID‐19 pandemic created a global public health crisis which disrupted the routine organisation and delivery of healthcare and affected the mental well‐being of nurses internationally (Maben et al. 2022; Moynihan et al. 2021). In the United Kingdom, healthcare was rapidly re‐organised early in the pandemic with non‐emergency (elective) healthcare postponed and urgent care re‐organised around infection control measures to deal with the rising number of people with COVID‐19 requiring critical care. This brought challenges for healthcare staff, including difficult working conditions, depleted care standards and conditions, moral distress, emotional exhaustion and burnout (Maben et al. 2022).

Prior to the pandemic, there was a global shortage of nurses which has grown since the pandemic, from 6 million to over 13 million (International Council of Nurses 2023). Burnout is prevalent with 40%–80% of global nurses experiencing this due to lack of support (International Council of Nurses 2023). The pandemic heightened shortages, initially through changes in service delivery and staff redeployed, self‐isolating and shielding, to longer term problems of long COVID and the backlog of unmet care needs from the scaling back of non‐COVID‐related healthcare and the continuing circulation of COVID‐19. During the pandemic, there was a 25% rise in the number of nurses leaving their role, with the largest increase in people leaving in the younger age groups (under 45 years) (Kings Fund 2021).

Previous research on the mental well‐being of nurses has focused on the experiences of nurses working in the USA (Kim et al. 2021) or England (Couper et al. 2022) or healthcare workers in Italy (Di Tella et al. 2020) and Australia (Holton et al. 2021). Scotland has a separate healthcare system from the rest of the United Kingdom, and little is known about the experiences of nurse's working in Scotland. As far as we are aware, no research to date has explored the experiences of nurse's emotional management while working during COVID‐19 pandemic. We set out to explore how the rapid reorganisation of the delivery of healthcare affected nurses' mental well‐being and their emotional management in one health board in Scotland.

## 2 Background

Nursing involves intensive emotional work, through recognising emotion in others and through managing one's own feelings and emotions, and presenting different faces or masks depending on demands (Bolton 2001). There are three types of emotional management in nursing: therapeutic, collegial and instrumental (Theodosius 2008). Therapeutic refers to interactions between nurses and patients and/or their families. Instrumental emotional management refers to their skills and confidence in communication while performing technical clinical processes or procedures. Collegial emotional management refers to the interactions and interpersonal relationships between nurses and their colleagues (Theodosius 2008). When emotions in interactions are genuinely felt, there is a sense of connection, job satisfaction and good patient experiences. When the required display of emotion is not aligned with genuine feelings nurses are at risk of stress, burnout and ill health affecting the quality and safety of healthcare (Schmidt and Diestel 2014; Delgado et al. 2017).

Nurses have agency and are skilled at emotionally juggling different feeling norms, alongside balancing clinical guidelines and organisational issues with individual patient need (Bolton 2001). Riley and Weiss (2016) argue emotional work can be rewarding for healthcare staff when they deliver care in different or additional ways to what is institutionally required (Riley and Weiss 2016). The management of emotion is characterised as tacit and not recognised or valued in healthcare (Delgado et al. 2020) which makes it vulnerable to displacement (Smith and Gray 2000).

There has been a hiatus in research exploring the emotional nature of nursing and little exploration in challenging conditions of interaction (Theodosius 2008; Gray 2010; Bolton 2001). The conditions of interaction challenge emotions as they are highly contextual, highlighting the importance of time and space to the emotional management literature (Dowrick et al. 2021). We present analysis of nurse's emotional management during the COVID‐19 pandemic drawing on Goffman's (1959) dramaturgy, a theorisation of social interaction.

Goffman's (1959) dramaturgy underpins much of the emotional management literature (Goffman 1959). Goffman theorises human interaction is like a grand play, where people create, maintain and destruct shared understandings of reality through working individually and collectively to present a common understanding of reality. These interactions are shaped by implicit rituals and understandings, learned through socialisation and create social order. Goffman's analysis of social interaction makes an important distinction between when people are performing front and backstage. Frontstage actors must perform in ways which are socially desirable, but may not reflect their true feelings, to achieve the desired audience response. Backstage performances actors can temporarily relax and drop their performance, as there will not be members of the audience present. We also recognise emotion as influencing relationships in healthcare and that people can influence and be affected by one another and wider societal orders and/or influences (Ahmed 2004).

It is important to understand how the reorganisation of the delivery of care during the pandemic affected nurse's mental well‐being and their ability to manage their emotions and the emotions in others, to reduce stress and burnout, and improve recruitment and retention. Given the hiatus in research within the emotional management literature in nursing, there is a need for more current literature. Furthermore, emotional management of nurses within challenging conditions and depleted care standards is underexplored. Emotional management is important for high quality and safe healthcare, patient satisfaction, clinical outcomes and retention of nurses (Serrano‐Ripoll et al. 2020; Bell and Wade 2021).

## 3 The Study

### 3.1 Aims

To explore the impact of the COVID‐19 pandemic on nurse's well‐being, experiences of delivering healthcare within acute settings and their emotional management.

## 4 Methods/Methodology

### 4.1 Design

A sequential mixed methods study comprising an online survey including validated health and well‐being scales in Jisc online surveys, followed by in‐depth interviews with a purposive sample of survey respondents. A mixed method approach was adopted to illuminate the experiences of nurses during the pandemic, using the quantitative data to sample, validate the qualitative data and provide a more holistic picture.

### 4.2 Theoretical Framework

We used Goffman's (1959) dramaturgy, a theorisation of social interaction and emotional management as a theoretical framework applied abductively.

### 4.3 Study Setting and Recruitment

Study participants were recruited by responding to an advert containing a link to the survey, disseminated in one NHSScotland health board via social media and the health board's COVID daily news update between February and May 2021.

### 4.4 Inclusion Criteria

Registered nurses working in secondary care during the COVID pandemic in one Scottish Health Board. Nurses were excluded if they worked in a community or hybrid setting due to resource constraints.

### 4.5 Data Collection

The survey was originally developed based on learning from another study exploring well‐being, (Douglas et al. 2021) and was piloted among academic nursing staff. It contained questions to determine the respondents' demographic profile, and mental well‐being and resilience scores. Mental well‐being was measured using the Warwick Edinburgh Mental Wellbeing Scale (WEMWBS) and resilience was measured using the 10 item Connor David Resilience Scale (CD‐RISC) (Cohen, Kamarck, and Mermelstein 1983; Wetherall et al. 2018). The WEMWBS is a 14‐item scale of subjective well‐being and psychological functioning with a score range from 14 to 70. Higher scores suggest better mental well‐being. The Scottish population mean score was 49.8 in the 2019 Scottish Health Survey (Scottish Government 2023). The CD‐RISC‐10 contains of 10 statements and total scores range from 0 to 40, where lower scores suggest less resilience, or more difficulty in bouncing back from adversity. CD‐RISC‐10 has been widely used worldwide and a general population sample of 18–34 year olds in Scotland reported mean score of 29.6 (Wetherall et al. 2018). The survey contained questions to determine the respondents' future career intentions, demographic profile and mental well‐being and resilience scores.

Twenty in‐depth interviews were conducted by RO and AG (experienced qualitative researchers) to gain an in‐depth understanding of nurse's experiences of working through the COVID‐19 pandemic, exploring the impact of rapid reorganisation of care delivery in complex and challenging conditions on their mental well‐being. Interviews were conducted and recorded on Microsoft Teams using video with purposively sampled questionnaire respondents who indicated they were willing to participate in an in‐depth interview (*n* = 108). Twenty were sampled to give a detailed picture. The sampling approach aimed for maximum variation in those redeployed, remained in usual role and returned from retirement, those in COVID and non‐COVID facing roles and by their stress and well‐being responses to the questionnaire. The interviews were conducted between April and July 2021, which was 13–16 months after the first UK government lockdown and as it was looking like the United Kingdom was about to go into a ‘third wave’ of the pandemic. A topic guide was informed by the literature and a previous study by the team which explored the experiences of healthcare students rapidly deployed in wave one (Douglas et al. 2021). Interviews lasted between 60 and 95 min and were digitally recorded and transcribed verbatim.

### 4.6 Data Analysis

Online survey data were analysed descriptively using the IBM Statistical Package for Social Sciences (SPSS; v25). Qualitative analysis was iterative with data collection. After familiarisation, an initial coding frame was developed based on a priori issues and inductive themes in the data, this developed through discussions with the whole team. All data were coded in Nvivo 20 and the framework approach was utilised for in‐depth analysis across emergent themes and characteristics and across the concepts and ideas within the nursing emotional management literature (Ritchie, Spencer, and O'Connor 2003). Emotional management did not inform the original methodology but as data gathering progressed, it became clear it was going to be a central lens with which to interrogate the data. An abductive approach to analysis was undertaken, revisiting the data once sensitised to this theoretical approach (Timmermans and Tavory 2012).

### 4.7 Ethical Considerations

Completion of the questionnaire was considered consent for the questionnaire and verbal consent was collected prior to the interview, adhering to social distancing at the time. This study was approved by Robert Gordon University School of Nursing, Midwifery and Paramedic Practice Ethics Committee (ref 21–03).

### 4.8 Rigour

The survey used validated health and well‐being scales (Warwick Medical School 2023; Cohen, Kamarck, and Mermelstein 1983). Reporting of the interview data adheres to the principles of qualitative rigour (Lincoln and Guba 1985). We completed the Good Reporting of A Mixed Methods Study (GRAMMS) checklist (O'Cathain, Murphy, and Nicholl 2008).

## 5 Findings

We initially present the results of the survey which provides mental well‐being scores followed by in‐depth qualitative analysis of participant's experiences of emotional management.

### 5.1 Survey

#### 5.1.1 Characteristics of Participants

Of the 108 who completed the survey, 44 (41%) were redeployed within the NHS due to the COVID‐19 pandemic, 102 (94%) described themselves as female and 79 (73%) reported they worked in adult nursing with 73 (68%) working full‐time. Characteristics of survey respondents can be found in Table 1.

**TABLE 1:** Characteristics of the online survey respondents (*n* = 108).

	*N*	%
Area of clinical practice		
Adult/general	79	73
Children	19	18
ANP or specialist nurse	8	7
Other (midwife, mental health)	2	2
Time since graduation		
Less than 1 year	1	1
1–5 years	15	14
More than 5 years	90	85
Current work hours		
Full time (37.5 h/week or more)	73	68
Part time (21 h or more)	28	26
Part time (less than 21 h/week)	6	6
Clinical area		
Hospital	98	92
Community	4	4
Other (both)	5	5
Gender		
Female	104	94
Male	6	6
Age group		
Under 25 years	11	10
26–35 years	31	29
36–45 years	19	18
46–55 years	31	29
56 years or over	16	15
Ethnicity		
White	103	95
Black, Asian, Minority Ethnic group	5	5

The mean mental well‐being score was 46.6, (SD 8.6) indicating they had lower well‐being scores than the general Scottish population (mean 49.8, [Scottish Government 2019] *n* = 24 (22%) had scores of 40 or less indicating ‘*probable depression*’; 25 (23%) had scores of 41–44 indicating ‘*possible depression*’ Warwick Medical School (2023)). So overall, the mental well‐being scores indicate that 45% of nurse respondents reported *probable or possible depression*. The mean resilience score was 27.0 (SD 6,4) and lower scores suggest less resilience, or more difficulty in bouncing back from adversity. During the pandemic, most participants indicated they found their job moderately stressful. Mean (average) stress scores are significantly higher than population norms in all age categories. These data are presented in Table 2.

**TABLE 2:** Mental well‐being, resilience and perceived stress scores.

Warwick Edinburgh Mental Wellbeing Scale	
Mean (SD)	46.61 (8.7)
	*N* (%)
Probable depression	24 (23)
Possible/mild depression	25 (24)
Unlikely depression	57 (54)
Connor Davidson Resilience Scale, mean (SD)	27.02 (6.4)
Perceived stress, mean (SD)	8.79 (1.3)

*Note:* Warwick Edinburgh Mental Wellbeing Scale (WEMWBS) is a 14‐item scale of mental well‐being. The scale scores 14–70. WEMWBS has a mean score of 51.0 in general population samples in the United Kingdom with a SD of 7. A score of 41–44 is indicative of possible/mild depression; a score of < 41 is indicative of probable clinical depression. Connor David Resilience Scale (CD‐RISC‐10) total score range from 0 to 40. Higher scores suggest greater resilience and lower scores suggest less resilience, or more difficulty in bouncing back from adversity. General population sample Scotland mean 29.6 (Wetherall et al. 2018). Perceived stress scale (4 item): Range scores 0–16; higher scores correlated to more stress.

Almost a third (32%) of respondents had thought about leaving nursing frequently or all the time in the past year. Fifty‐nine (56%) reported being unlikely to leave nursing or explore other career opportunities and 33 (31%) reported being unlikely to leave nursing *but* likely to explore other career opportunities. Sixty‐two per cent intended to continue with their current job or to stay working within the health board and only 7% wanted to move to a different NHS organisation.

### 5.2 Qualitative Findings

#### 5.2.1 Characteristics of Participants

Twenty participants were included in the qualitative component, most were aged between 26 and 55 years and working from grades five to eight (five nurses at band 5, two at band 6, 12 at band 7 and one at band 8). Seven were redeployed into a COVID ward, 11 were working in a COVID ward and two were in managerial positions where they worked remotely. For all participants, the COVID pandemic was a period of uncertainty and disruption. We now present our qualitative analysis showing working through the pandemic was emotionally demanding and emotional management was impeded by infection control measures. First, we explore the importance of their professional identity in driving their decision to contribute to the pandemic effort despite fearing the consequences of infection.

#### 5.2.2 Professional Identity and the Moral Imperative to Be Seen Contributing

Most participants felt that there was a moral imperative to be seen contributing to the pandemic effort, despite the risk this posed to themselves and their family members. There was a strong sense of nursing identity. One senior nurse said there was also moral imperative to been seen to be contributing alongside their team.

‘I could bring that (COVID‐19 infection) home to someone that I'm living with who's shielding…But then, my moral compass, I suppose, was that I can't be at home…I can't ask my team to do something that I'm not prepared to do myself…’. (Beth)

Although it was not an easy decision to make, their professional identity was reported as the main driver of their decision‐making, despite the risks it was their professional duty as a nurse to put others before themselves.

‘I'm very much to the mindset like I'm a nurse, I will do whatever I need to do in job…I'm going to do it, regardless of my own safety. Because, as a nurse, you put your patient first’. (Noah)

Furthermore, some of the infection control measures were perceived to be inhumane and challenged their identity as a nurse as explained in the following section.

#### 5.2.3 Frontstage: Interactions With Patients

Here, we present qualitative data of the participants experiences of front of stage performances of delivering care during the COVID‐19 pandemic.

##### 5.2.3.1 Barriers to Communication and Expressions of Empathy

For those working in settings with positive COVID‐19 patients, infection control measures significantly impacted upon interactions between patients and nurses. Personal, protective equipment (PPE) inhibited communication, the wearing of masks and visors was seen to affect the quality of communication with patients as they could struggle to hear, or lip read. The wearing of face masks, at critical stages, such as at end of life or when trying to talk to patients with communication difficulties, was also perceived, by some, to interfere with the expression of love, sympathy and empathy for patients and relatives.

‘You want them to be able to see all your facial expressions and, you know, know, let them know that you're there for them or whatever. I think that (PPE) is a big barrier when it comes to nursing. You know, we just, you know, just being able to show some sympathy, empathy, love, you know, all the rest of it. I think you can't always get that over, can you, when you're wearing a mask and a visor and all the rest of it?’ (Rhona)

PPE inhibited communication with patients who were deaf, not proficient in English or with learning disabilities or a mental health condition.

‘Yeah, it's difficult, especially if the patient's deaf or, you know, not English speaking, or, you know, it's very difficult to communicate…, but they can't see my face now. So, it's difficult’. (Eva)

For others, the inability to engage in emotional management caused some nurses to bend the rules and act with agency. One nurse describes an incident when she went against COVID rules to do what, she felt, was morally right for the patient.

‘I remember this lovely, poor little patient who was so demented, and she wouldn't open her eyes, she wouldn't even drink for anyone because we're wearing the masks. And I was like, oh, this is just bad. This is just bad, and I had to, at one stage, pull the curtain round, put my mask down and say please eat, please take a drink’. (Niamh)

The lack of patient‐centred care during the pandemic weighed heavily upon them with many struggling to switch off when at home.

‘…I would just be awake, like four or five hours every night…I think just worry about things like that, that were happening on the ward’. (Niamh)

##### 5.2.3.2 End of Life Care

On COVID facing wards, staff found themselves taking on additional roles usually undertaken by family members and sitting with patients while they died. This was difficult and compounded by wearing PPE which impacted upon expressions of empathy (as previous described).

‘Because there was no family or anything allowed in there…you're kind of watching people fading away, and all they're seeing is somebody they don't know, with a mask over their face holding their hand really. Yeah, that's quite difficult’. (Shona)

##### 5.2.3.3 Clinical Expertise

Participants were unprepared and inexperienced in the care of patients with COVID 19 infection and those on non‐COVID‐19 wards were nursing outside of their speciality. The virulence of the disease, the fast deterioration of patients, volume of severely ill patients and high mortality rate was frightening and distressing. The speed and frequency with which someone could die from COVID‐19 took some participants by surprise and they felt unprepared to give end‐of‐life care, including patients who died on their own, without the care and companionship of loved ones at the end of life.

‘…the deterioration in COVID patients happened literally within minutes…And that's something that, that you weren't really told…you were never really made aware of how quick a COVID person can deteriorate, which was the scary thing’. (Noah)

The inexperience of dealing with the disease also resulted in a lack of confidence and ability in clinical skills, dissatisfaction with care standards and stress.

‘…on top of having a pandemic, on top of doing something you'd never done before, was just a lot of stress, a lot of stress and I then, I didn't sleep very well…because you've been deployed into something that, that you don't really, you don't, you feel like you don't really know what you're doing….but sometimes you almost felt a bit more of a hindrance, because you felt out of your depth’. (Beth)

In these situations, the wearing of a mask was useful. Staff were able to hide behind their masks and use the masks to hide their emotions.

##### 5.2.3.4 Interacting With Patients on Non‐COVID Wards

In wave one of the pandemic routine care was cancelled and many wards specialised in the care of COVID‐19 patients, the ‘non‐COVID’ wards had to take all other patients. Initially staff were on standby and felt guilty for not being on the frontline; however, these wards gradually got busier. At the time, the UK public were on their doorsteps on a Thursday evening and clapping and displaying rainbows in support for NHS staff. These staff expressed a sense of loss from colleagues redeployed, demoralised, discomfort for not matching public perceptions and experiencing a useless and helpless decline in care standards for non‐COVID patients.

‘…even patients who were coming into hospital…had their care reduced…these were people who suffered because of the pandemic’. (Niamh)

By wave two, non‐COVID wards were much busier than in wave one. Staff felt inexperienced and unprepared for caring for patients outside their usual clinical discipline. Some participants were dealing with a high number of Child and Adolescent Mental Health Services (CAMHS) patients:

‘…we were getting more of a medical, medical side. So, things like diabetes and head injuries and things like that we wouldn't have normally of seen. The biggest one probably would have been the CAMHS patients, there's a lot of them that we weren't used to’. (Duncan)

One nurse describes how experiences of inadequate support when caring for conditions with which they had no clinical experience had affected her and her team:

‘Our Ward Manager…devised like a folder of how to care for certain illnesses and conditions that we were unused to…There was a lot of anxiety amongst the staff… It led to quite a low morale throughout (the pandemic)’. (Eilidh)

Some in non‐COVID facing roles also experienced emotional distress in the form of guilt from being redeployed from COVID facing areas.

‘…you're thinking to yourself, well, maybe, you know, “I should be down in ITU.” (I had) a sense of being sort of underutilised… One: I felt like I'd let the side down hugely and two: I felt like a spare part’. (Shona)

Infection control measures affected the ability of nurses to manage emotions in patients. On occasion, moral distress at reduced patient‐centred care caused some nurse to bend the rules to ease their distress. Nurses also had to give a performance of confidence in their clinical skills, where most nurses were caring for patients with which they had little or no experience. Their emotional management performance was extended in caring for patients with no family around, taking on additional care usually provided by families, which was most distressing at end of life.

##### 5.2.3.5 Interactions With Families

Visitors into hospital were not allowed and later restricted, separating patients and families at a time of need. This expanded the emotional management of nurses in undertaking the support of patients usually undertaken by families and in supporting families remotely. Nurses had to stop family members from coming into hospital and had to communicate with them over the phone.

‘…because relatives couldn't come in and be with them, like we, like they were able to before. Erm, speaking to them over the iPads, I think we probably had, probably had a lot more relative involvement over the, over the iPads than what we, we probably would have done face‐to‐face…’. (Noah)

For end‐of‐life care in COVID and non‐COVID wards, staff had to facilitate final conversations between patients and their family members. In wave one, they were using tablets, and by wave two, restrictions were lifted slightly and family members were allowed into the hospital for short periods of time.

‘And listening to their, to them, having that last conversation wasn't nice, and it's, so probably something we probably wouldn't hear normally…’. (Noah)

On the non‐COVID ward, participants talked about the mental distress among family members separated from their loved ones in hospital. This seemed particularly acute on the neonatal ward where new parents had been separated from their baby and family.

‘…it's the neonatal unit, so, the parents are anxious anyway. But of course, this added to that, and then the lack of support that they had from friends and family members due to the visiting being restricted’. (Gillian)

Staff found implementing of checking COVID status very distressing. Swabs were taken to detect for COVID and results took 48 h to come back which is a long time to wait to see your baby.

‘…you know, that was up to 48 hours, sometimes. Erm, which, you know, is a crazy amount of time to be told that, actually, you know, you feel fine, but you can't come and see your baby’. (Gillian)

And

‘…you were speaking to parents on the phone, you know, who couldn't see what you were seeing, you know, you'd done the first nappy change, you'd heard them cry, you know, they, that's all the firsts that they've missed out on’. (Gillian)

Implementing the infection control measures also weighed heavily upon staff in COVID wards:

‘…the rules were nobody was allowed to visit or one person could come visit for one hour, you know, I just found that, that, to me, as a nurse, it just made me question my whole ethos as a nurse…this is something I've never had to do before, stop somebody coming in to visit their dying relative, it was just awful…’. (Niamh)

Also issues with connecting with families also caused distress:

‘We knew our patient was going downhill…you were getting the dread of having a phone their relatives to let them know what's happening, and then, you know, particularly elderly patients who, who might not have a smartphone or an iPad who would be able to, to use this technology’. (Noah)

The separation of patients and families generated additional emotional work for nurses on both COVID and non‐COVID wards. The work of preventing family members from being with their relatives and facilitating final conversations caused moral distress requiring additional work in managing their own emotions while interacting with families.

##### 5.2.3.6 Interactions With Colleagues

Staff team structures were very different in COVID and non‐COVID wards, with additional staff nurses in COVID wards and reduced nurses in non‐COVID wards, so they were not working with their usual team dynamics. Interactions with colleagues were reduced on COVID‐facing wards, social distancing measures meant nurses worked for long hours, often a whole shift, isolated from their colleagues. Communal space and/or managerial offices and senior staff were removed for the donning and doffing of PPE and breaks were staggered for social distancing. Non‐COVID wards lost many nurses through redeployment, and in some areas, such as oncology, they were often managing the same caseload with a significantly reduced workforce. Nurses were experiencing exhaustion from the physical and emotional work.

‘…we were doing everything, you know, where the work is normally divided between five or six of us, it was kind of two of us doing all the work’. (Isla)

On the COVID wards, boundaries were created between those who were on the floor, caring for COVID patients (grade fives) and those in more senior management positions. Managers were often moved away from clinical areas to create more space for COVID‐19 patients or for the donning and doffing of PPE. This meant they were rarely in reach to ask for help, which was stressful for junior nurses managing complex cases alone. COVID‐19‐positive patients in need of intensive care or high dependency could only be cared for by one nurse who was usually located in a private room. Participants who had been in those rooms described how alone, frightened and distressed they were.

‘…we were taking two patients ourselves, you know, ventilator patients, which is not supposed to happen, and my managerial staff just weren't, weren't here. And they have the experience to look after these, these sort of patients…that was frustrating’. (Ailsa)

And

‘So, there were quite a few blowouts, and situations on occasions, obviously, nothing in front of patients or public or anything like that. But the stresses that I would feel like between band fives and band sixes, the sense of hierarchy, and people not doing their bit…’. (Hannah)

By the end of the second wave, grade seven nurses were back working alongside their grade five and six colleagues, caring for patients but tiredness and staff sickness and absences were starting to rise.

‘…people would go off sick, stress leave, all of that…and then, you know, the immense, you know, the strain that that puts on the rest of the team and how, and how that plays into the, the dynamics, and the tensions of working on a daily environment…’. (Hannah)

Although removed from the frontline, managers were stressed and trying to keep up‐to‐date with the constant changes from government and health board, redesigning pathways and care procedures appropriately and communicating these changes to staff. Senior nurses reported they struggled with the weight of expectation from their staff, feeling that their team expected them to know everything, and they did not as things were so uncertain. This led to a disconnection.

‘I felt guilty that I wasn't there as much as I would like to be because of other things that pulled me away. And I know that, it's really hard when, and I think there was almost like a, a disconnect between management and the clinical workers’. (Beth, Redeployed to a Senior Management role)

For both COVID and non‐COVID wards, there was stress and tension from teams working with less staff and not their usual staff.

‘…the whole team was just completely annihilated, erm, just everybody was just dispersed, and you just felt so, erm, so on your own’. (Isla, non‐COVID ward)

For some who were working on mixed wards (with COVID and non‐COVID patients), there was more informal collegial support as team structure did not change to the same extent.

‘Old and new faces, I found that they were the support, because we're all doing the same thing. You could quite happily vent your, your feelings to these people, and they knew exactly what you were going through, and they support, like, we all supported each other’. (Noah)

There were strong feelings around a lack of emotional support from management: Some nurses returned who to work after a COVID‐19 infection reported their line manager did not ask how they were and nurses who had childcare responsibilities reported a lack of flexibility when childcare facilities were closed, and grandparents were shielding.

‘…I cannot start, you know, then because there's no childcare. I am single, you know, with my two boys, I don't, you know, have family around. So, there was this back and forth, you know, exchange of words with my team leader…’. (Adah)

The reorganisation of nurse staff levels and social distancing measures and the physical and emotional work of nursing through the pandemic affecting staff led to a loss of connection between colleagues. Working in different teams and in isolation for some had a profound impact. As the pandemic progressed staff not only experienced burnout and stress, creating tensions within teams but also leading to sick leave and thus putting more pressure on stretched teams.

#### 5.2.4 Backstage Stage: Managing Emotions

Working through the COVID‐19 pandemic created several emotions for nurses including, fear, isolation, anxiety, stress, guilt, anger, frustration and moral distress (through being dissatisfied with the care they were providing to their patients in both COVID and non‐COVID facing roles).

‘I definitely think I have been a lot more stressed than I probably ever have been, during this pandemic’. (Eilidh)

They felt exhausted too:

‘I just feel like it's taken every bit out of me, you know, just taken everything’. (Rhona)

Approximately a third of our sample qualitatively reported mental health problems which emerged during the pandemic that had affected their functioning at work and home. With anxiety the most reported. Participants with pre‐existing mental health problems, that had been well managed pre‐pandemic, anticipated that COVID‐19 was likely to take a toll on their health. Some were able to seek appropriate support, whereas others felt the inflexibility of shift patterns re‐triggered post‐traumatic stress disorders and issues with alcohol consumption arose. Staff who had experienced a mental health problem for the first‐time perceived stigma from colleagues, including line managers, and as a result were reticent about disclosure.

‘I have got Post Traumatic Stress Disorder (since 2014), which I live quite comfortably with and function pretty well with…. but I went from having zero panic attacks for, you know, at least a couple of years to having maybe one a week. They've gone from being non‐existent to being kind of pretty normal now, since last year’. (Shona)

And, another who had never had a mental health problem prior to the pandemic, developed an anxiety disorder.

‘I'd never been anxious in my life…I literally took to my sofa– I got in my car one day and couldn't drive to work’. (Niamh)

Upon describing their emotions, we now illustrate how participants managed their emotions.

#### 5.2.5 Personal Emotional Management Strategies

Most participants had coping strategies which they were used to drawing upon but many of these strategies were not available at the time due to the national lockdowns. Many participants struggled to switch off from work during their time off, nowhere to go and constant media coverage. Many had to stop watching the news to help create more of a divide between work and home.

‘I stopped watching…normal TV altogether…sticking to things like Netflix and that because I knew that, that wasn't going to be interrupted by an announcement, or, you know, this is what's changed, or these are what the numbers are today’. (Gillian)

The commute to and from work was useful for others to switch off from work and home:

‘I live a little bit out of town so, you've kind of got a half hour drive in, which is your, kind of getting your head into work mode bit, and then you've got your half hour driving home where you have a chance to just kind of chew it over in your head what happens but then, you know, it's, it's crossing the threshold at your home and discarding the last of your work gear that is kind of that's your, that's my switch off’. (Mairi)

The situation at the time also made it difficult for some nurses to engage in their usual personal emotional management strategies.

‘I lost a lot of my outlets for my stress, because I had the gym, well, that had gone, and cinema, that had gone. And I usually visit my family four or five times a year down in England, and that's all gone’. (Ruby)

Many managed their emotions through talking with friends and family:

‘I've got good friends that I can talk to. I've got my husband, got my kids. So, yeah, I've got, just talk. I'm a talker, so I just don't keep things bottled up, I just let it out. Have a good cry now and again, and then that gets, get you on to the next step. So, it's fine’. (Eva)

At the time of the interviews, there was talk of a third wave and this was devastating for our participants. They had felt that if they worked hard enough the pandemic would be over and they indicated that they were going to struggle to muster the energy to keep going.

‘…really felt like there wasn't going to be a light at the end of the tunnel…and this was going to be my experience for the next two years…just going to work, going home at night sleep, eat repeat’. (Hannah)

##### 5.2.5.1 Caring for Each Other

Pre‐pandemic nurses first strategy for managing difficult emotions was through interacting with each other, often through humour but also just a hug or a chat:

‘…we've got, as nurses, we've got that, that dry, dark humour… it definitely helps when you're in a situation that you're really struggling with. Some people come away with a just a off the cuff comment, and that would, that would be everybody. Just increases really’. (Noah)

On COVID wards, and to a lesser extent on non‐COVID wards, the reorganisation of care and social distancing measures challenged nurse's ability to interact and care for each other as described above.

‘…it would've been great on the ward, if we'd had the ability, you know, to be able to go somewhere, or just, or sit together and discuss how we're feeling and what we think's going well, and what we think could be improved, things like that’. (Rhona)

There was limited backstage space to remove their masks and empathise and look after each other through what they were witnessing and masking while out front of stage.

‘It was, it was hard, you know, because often…you wanted physical contact from your colleagues, you know, that that support you get normally, you know, putting your arm around somebody and saying, it's all right’. (Mairi)

Some nurses eased the social distancing rules to care for colleagues they could see were visibly upset.

‘I remember one day… one of the domestic staff, and she was just crying and crying…I took her outside. And she says, oh, I just need to get my mask off. I was just so panicked…wouldn't it have been nice if she'd just had that opportunity just to go somewhere, just to breath for five minutes…’. (Niamh)

Throughout their careers, participants had developed strategies to support one another to manage difficult emotions. The reorganisation of care, redeployment, social distancing measures had unintentionally removed many of these opportunities to care for each other. There was a real sense of loss and suggestions that some may have coped a bit better with their usual forms of collegial support.

#### 5.2.6 Psychological Support Impact of COVID‐19

Psychological support was provided by the health board in the form of a well‐being hub, psychologist available in staff rooms and online support. These services were used by a minority of our sample. The psychologist at lunchtime was perceived by some as not the most appropriate time:

‘…there was a psychologist that came in and came into the staff room during breaks but…we were only getting three half an hour breaks a day. We didn't want to be spending that time speaking to someone, you know, we were trying to get our lunch down our throats… it's not very personal’. (Ailsa)

The hub had to be accessed outside of working hours which was perceived as a barrier:

‘Yeah, yeah, there was the psychology hubs. But after a day of it, you just, you wanted to go home, or I wanted to go home…kind of forget about it a bit not go over?’ (Isla)

Some of those who accessed these services found them useful, whereas perceptions of stigma did influence engagement for others:

‘I did take advantage of the counselling service that the hospital offered. So, they listened to me with what I was struggling with at the time…that was that was good’. (Rhona)

And

‘…there was only ever three of us…you know what we're like, nobody likes to admit anything's bothering them in this place…there's still a bit of a stigma that comes with mental health issues, and I didn't want them thinking, you know, she's a nutter. So, you just kind of went through the motions of sort of going in and sitting down and, you know, listening, as opposed to talk and really’. (Shona)

There appeared to be a culture of being stoic among nurses which may have prevented engagement with services.

‘…they're a stoic bunch. They really, they know how to get on with it, they're very adaptable, because…it's a very, it's a very demanding work environment’. (Hannah)

For others, there was a feeling that they were not ready to try and process the pandemic while it was ongoing:

‘…I feel like almost reliving everything would be worse than just, you know, just keeping going and, and we're not even out of it yet’. (Adah)

Nurses were working in extreme and unprecedented working conditions, managing many difficult emotions, with some managing better than others. Nurses were used to managing difficult emotions through interacting with colleagues and caring for one another. However, they felt disconnected from colleagues, working in teams with different structures and dynamics, with social distancing measures preventing them from engaging as they did pre‐pandemic. Furthermore, it was difficult to switch off with constant media attention at the time and many of their personal coping strategies closed, such as the gym. Some were able to cope through talking to friends and family. Talk of a third wave was affecting motivation and as the pandemic continued sick leave and tensions were rising. Psychological support was provided and utilised by a minority but there was a culture of being stoic and staff feared stigma.

## 6 Discussion

Nursing through the pandemic was emotionally demanding, requiring greater and harder emotional management performances front of stage. Nurses experienced a wide range of emotions through working in challenging conditions and unprecedented circumstances. Our analysis highlights a loss in connection with patients, their families and colleagues through the reconfiguring of space, social distancing and PPE making the work of emotional management harder. They also had additional emotional work caring for patients and their families. They witnessed death, suffering and patient centred care compromised while performing front of stage. They performed clinical confidence despite lack of clinical skills and experience. The relentless nature of working through the pandemic was emotionally exhausting which made maintenance of their performance harder, especially as there was talk of wave three of the pandemic. Furthermore, our mental well‐being scores indicated that nearly half our sample reported probable or possible depression and had lower resilience scores suggesting low mood and difficulty in bouncing back from adversity, suggesting emotional exhaustion and burnout, which also makes the front of stage performance harder to perform.

Participants were used to working in emotionally challenging situations; however, infection control measures and the reorganisation of care impeded access to some of their usual emotional management strategies. Nurses had previously relied on backstage space and interactions with colleagues, through humour, hugs and conversations about difficult situations; however, social distancing measures and the reorganisation of space and reduced these opportunities. Participant's missed collegial support highlighting the importance of creating time and space for this in future. These are similar findings to those of Dowrick who explored emotional management in healthcare workers in wave one; however, Dowrick did not find the lack of support for one another and did not have mental well‐being scores to complement their qualitative analysis (Dowrick et al. 2021). The emotional management literature which highlights the importance of backstage for processing experiences ‘frontstage’ and caring for one another (Bolton and Boyd 2003).

The disconnect between management and the lower grade nurses directly caring for patients and between nurses on COVID and non‐COVID wards created an emotional divide, tensions and grievance. There was a real lack of connection, understanding and empathy through a lack of shared space and opportunity for communication and compassion. Ahmed's (2004) work is seminal in showing how emotions influence boundaries between people, arguing ‘emotions do work to align some subjects with some and against others’ (Ahmed 2004, 117) The pressures the NHS was under with the need to respond rapidly to virulence of the pandemic meant they underestimated the organisational and emotional value of backstage spaces to processing and developing emotional management. Our findings indicate almost a third are thinking about leaving their profession and connections with colleagues have been found to improve retention of nursing staff (Fisher et al. 2022).

The discourse during the pandemic morally reframed behaviours to promote compliance with public health and government messaging. Combined with the organisational directives of strict infection, control measures defined the emotional agenda, removing opportunities for agency and creating emotional unintended consequences. Emotional distress did cause some nurses to bend the rules and resist the strict rules at the time to do what they felt to be morally right for the (particular) patient. Some bought into the necessity of the infection control measures and these were easier to implement. However, for those where these rules challenged their nursing identity, it led some to question their profession and whether they could continue to work as a nurse. They also described high emotional costs unable to detach themselves from work when not working similar to Johnston's findings of emotional work in a care home (Johnson 2015).

### 6.1 Strengths and Limitations

A strength of this work is that we were able to capture in‐depth experiences of nurses in COVID and non‐COVID facing roles, those of different grades, including clinical managers and across waves one and two of the pandemic. A further strength is the mixed methods study design which used validated mental health and well‐being scales to compare our study population, adding validation to their qualitative reports of poor psychological well‐being. The richness of the qualitative data does however, shadow the quantitative findings. Use of Goffman's dramaturgy and emotional management literature as a theoretical framework has added greater depth to the analysis and illuminated the importance of backstage space for peer support.

A limitation of a study is that it was conducted in one NHS Scotland health board, although the findings do align with similar studies from other countries and settings (Ashley et al. 2021; Maben et al. 2022; Liu et al. 2021; Liberati et al. 2021). Our sampling strategy and data gathering via Microsoft Teams may have limited how representative our study participants were of people working in acute care at the time; however, this was the only way to interview people at the time due to the government restrictions. The experiences of those working in the community is missing. Most of the quantitative and qualitative participants were female which is representative of the gender distribution of the nursing workforce. As they were self‐selecting, we may have been more likely to capture the views of those more aggrieved. Black, Asian and minority ethnic groups were under‐represented in the sample.

### 6.2 Recommendations for Further Research

Recommendations include designing and developing interventions which recognise and support emotional management. Educational interventions delivering emotional intelligence have been found to improve emotional intelligence and may be transferable to address emotional management (Nelis et al. 2009). Our findings highlight a special kinship among nurses caring for one another in emotionally challenging situations. Furthermore, formal psychological intervention was only utilised by a minority of our sample. Future strategies may wish to explore peer support as a potential intervention and address cultural barriers, such as perceptions of being stoic. The experiences of patients and family members is under‐represented in the literature and may provide useful insights.

### 6.3 Implications for Policy and Practice

Healthcare organisations should consider how the re‐organisation of care influences interactions and emotional management. There is a need for better recognition of emotional management work and for the importance of collegial support in processing emotions generated through challenging work and conditions. These caring interactions should be prioritised in future. There is a need to dedicate time to offstage interaction in caring for one another, especially in times when frontstage performances are harder to perform. In a future pandemic situation, health policy should recognise the potential additional work of nurses and the potential consequences for staff well‐being, patients, families and patient safety.

## 7 Conclusion

Nursing through the COVID‐19 pandemic in secondary care was emotionally challenging, requiring performances front of stage which were harder to perform. Nurses had to hide their fears, emotions and the death and suffering they were witnessing. Infection control measures resulted in a loss of connection with patients, families and colleagues adding to the weight of emotion requiring managing. Emotional work was extended to caring for patients and families. Emotional well‐being was poor also making the front of stage performance harder. Backstage space is important for emotional management, but infection control measures removed many opportunities making caring for one another harder to achieve.

## Author Contributions

A.G., R.O., F.D., C.K., D.B. and N.T. made substantial contributions to conception and design, or acquisition of data, or analysis and interpretation of data. A.G. and N.T. involved in drafting the manuscript or revising it critically for important intellectual content. A.G., F.D., C.K., D.B. and N.T. given final approval of the version to be published. Each author should have participated sufficiently in the work to take public responsibility for appropriate portions of the content. A.G., F.D., C.K., D.B. and N.T. agreed to be accountable for all aspects of the work in ensuring that questions related to the accuracy or integrity of any part of the work are appropriately investigated and resolved.

## Conflicts of Interest

The authors declare no conflicts of interest.

## Peer Review

The peer review history for this article is available at https://www.webofscience.com/api/gateway/wos/peer‐review/10.1111/jan.16563.

## Data Availability

The data that support the findings of this study are available from the corresponding author upon reasonable request.
